# Modeling the link between the plausibility of statements and the truth effect

**DOI:** 10.3758/s13423-025-02647-z

**Published:** 2025-03-04

**Authors:** Semih C. Aktepe, Daniel W. Heck

**Affiliations:** Department of Psychology, University of Marburg, Gutenbergstraße 18, 35032 Marburg, Germany

**Keywords:** Truth effect, Repetition, Fluency, Modeling, Bayesian statistics

## Abstract

People judge repeated statements as more true than new ones. This repetition-based truth effect is a robust phenomenon when statements are ambiguous. However, previous studies provided conflicting evidence on whether repetition similarly affects truth judgments for plausible and implausible statements. Given the lack of a formal theory explaining the interaction between repetition and plausibility on the truth effect, it is important to develop a model specifying the assumptions regarding this phenomenon. In this study, we propose a Bayesian model that formalizes the simulation-based model by Fazio, Rand, and Pennycook (2019; *Psychonomic Bulletin & Review*). The model specifies how repetition and plausibility jointly influence the truth effect in light of nonlinear transformations of binary truth judgments. We test our model in a reanalysis of experimental data from two previous studies by computing Bayes factors for four competing model variants. Our findings indicate that, while the truth effect is usually larger for ambiguous than for highly implausible or plausible statements on the probability scale, it can simultaneously be constant for all statements on the probit scale. Hence, the interaction between repetition and plausibility may be explained by a constant additive effect of repetition on a latent probit scale.

The repetition-based truth effect refers to a cognitive bias wherein individuals tend to perceive statements as more likely to be true when repeatedly exposed to them, even if the statements are, in fact, false (Hasher et al., 1977).^1^ This psychological phenomenon is often attributed to the ease of processing, where statements encountered multiple times are perceived and processed more fluently (Arkes et al., 1989; Unkelbach, 2007). Individuals are assumed to rely on processing fluency as a cue for judgment and decision-making, particularly in the absence of prior knowledge regarding the statements (Begg et al., 1992). The metacognitive experience of processing fluency is assumed to contribute to a positive evaluation, leading individuals to judge repeated statements as more likely to be true in comparison to newly encountered statements (Unkelbach et al., 2010; Reber et al., 1998; Winkielman & Cacioppo, 2001; Reber et al., 2004).

According to Dechêne et al. (2010), the truth effect is a robust phenomenon but occurs primarily when dealing with ambiguous statements whose accuracy is unknown. Put differently, people are more likely to believe statements that are unclear and about which they have limited knowledge, such as “Proxima Centauri is the closest exoplanet to the Earth.” In contrast, statements such as “Mars has rings around it,” an incorrect fact that people are likely to have knowledge about, are less prone to the truth effect after repetition. Data by Newman et al. (2020) support this idea by demonstrating that ambiguous statements, when repeated, are more susceptible to the truth effect. This suggests that the lack of clear knowledge or background information can amplify the distortion of truth judgments.

In this regard, the amount of prior knowledge emerges as a critical factor influencing the truth effect (Brashier et al., 2017). Pennycook et al. (2018) showed that individuals with more general knowledge are less susceptible to the truth effect, implying that a well-informed population may be more resilient to the impact of repeated misinformation and fake news. Thus, prior knowledge seems to act as a protective factor against the truth effect. This claim is also supported by the fact that the truth effect diminishes when individuals receive feedback about the actual truth status of statements (Brown & Nix, 1996).

Although many factors such as the formulation, context, and source contribute to the plausibility of a statement, the most prominent factor affecting plausibility is the degree of prior knowledge held by individuals. The decrease in ambiguity through prior knowledge seems to reduce the truth effect. However, Fazio et al. (2015) asserted that the truth effect still occurs even when people have prior knowledge. In their study, participants were presented with statements that are expected to be known or unknown. Following the typical experimental paradigm for studying the truth effect, participants had to rate the truth of statements after half of them were presented in the exposure phase. A model-based analysis showed that people relied on repetition as a cue, even though they knew the actual truth status of the statements (but see Schmidt & Heck, 2024).

Numerous studies have consistently shown that repetition enhances the perceived truth of statements even if their content contradicts individuals’ existing knowledge (Dechêne et al., 2010). The truth effect is robust across various scenarios and has been replicated for highly implausible statements (Lacassagne et al., 2022), implausible fake news (De Keersmaecker et al., 2020), and even for facts that clearly go against common knowledge (Brashier et al., 2017; Brashier & Marsh, 2020). Nevertheless, the question arises whether the *magnitude* of the truth effect depends on the plausibility of the statements.

To address this question, Fazio et al. (2019) built on fluency theory, which assumes that repeated exposure to statements increases the subjective ease of processing (Unkelbach, 2007; Reber & Schwarz, 1999). The authors defined plausibility as a latent construct inherent to a statement, which determines the probability of judging that statement as true. Based on fluency theory, the hypothesis was derived that repetition induces an additive effect on the perceived, latent plausibility of a statement. In other words, the repetition effect on plausibility is assumed to be constant for all statements, irrespective of their initial plausibility. To make predictions for binary truth judgments on a probability scale, Fazio et al. (2019) used a simulation-based model. Both the model and an experimental study showed that, when considering observable binary truth judgments, the truth effect, defined as the difference in average truth judgments for repeated and new statements, indeed has the largest effect size for statements with a medium level of plausibility (i.e., approval rates of about 50%). Compared to verbal theories, formal models provide more explicit representations of assumptions regarding the functioning and interaction of system components (Smaldino, 2017). In this regard, the methodology employed by Fazio et al. (2019) stands out in the truth effect literature. Their distinctive approach uses a computational framework with simulations to elucidate the assumptions and predictions derived from the fluency theory.

Fazio et al. ’s (2019) efforts contribute to theory development regarding the link between fluency and the truth effect. However, their approach presents challenges in terms of formal modeling and statistical model comparison. The core hypothesis is that repetition results in increased processing fluency, which has the same effect on plausible, implausible, and ambiguous statements. Nevertheless, this hypothesis lacks formal precision, since the link between plausibility and the size of the truth effect, which involves nonlinear transformations, is only implicitly specified via simulations. The necessary assumptions regarding the nonlinear functions are not made explicit, which diminishes a major benefit of formal modeling. Fazio et al. ’s (2019) statistical model evaluation is also problematic since the predictions generated by their simulation-based model are only indirectly compared against the experimental data. This makes it difficult to assess whether the model adequately describes the data.

As a remedy, we formalize and implement Fazio et al. ’s (2019) simulation-based model as a Bayesian model. We improve the statistical analysis by fitting the model to data. Thereby, we can directly compare the predictions of alternative mechanisms. In addition to reanalyzing the experimental data by Fazio et al. (2019), we fit our model to the data by Nadarevic et al. (2018) for further validation.

## Formal model specification

### Simulation approach

Fazio et al. (2019) employed a simulation-based approach to investigate the relationship between the plausibility of statements and the size of the truth effect. To illustrate the predicted patterns of different theoretical assumptions regarding this relationship, the simulation assumed that each statement *i* has a certain plausibility $p_i$. The generative model builds on the idea of simulating a very large number of hypothetical latent truth values $y^*$, which are inherently not observable, to compute the predicted probability of “true” and “false” responses for new and repeated statements.

The plausibility parameter $p_i$ spanned from $-1$ for highly implausible statements to 2 for highly plausible statements (with increments of 0.01). For each new statement *i*, 100,000 latent values $y^*$ were drawn from a normal distribution centered at $p_i$ with a standard deviation of 0.5. For repeated statements, the latent values $y^*$ were sampled from a normal distribution, which also had a standard deviation of 0.5 but was centered at $p_i + f_i$ with $f_i$ corresponding to the additive effect of fluency induced by repetition (depending on the specific model, $f_i$ may depend on plausibility). To obtain the predicted probability of judging a statement as true or false, the continuous latent scores with values between negative infinity and positive infinity were converted into dichotomous responses by assessing whether they are above or below the threshold of 0.5, respectively,

1 $$\begin{aligned} y^\text {new}_i&= {\left\{ \begin{array}{ll} \text {``true''}, & \text {if } y^* > 0.5 \text { where } y^* \sim \text {Normal}(p_i, \sigma )\\ \text {``false''}, & \text {otherwise;} \end{array}\right. } \end{aligned}$$

2 $$\begin{aligned} y^\text {rep}_i&= {\left\{ \begin{array}{ll} \text {``true''}, & \text {if } y^* > 0.5 \text { where } y^* \sim \text {Normal}(p_i + f_i, \sigma )\\ \text {``false''}, & \text {otherwise.} \end{array}\right. } \end{aligned}$$

Separately for different plausibility levels, Fazio et al. (2019) computed the relative frequency of “true” responses out of the 100,000 simulations as a proxy for the predicted probabilities for new and repeated statements. The authors explored various assumptions regarding the relationship between the size of the fluency effect ($f_i$) and the initial plausibility of statements ($p_i$), including cases where the effect is independent of plausibility or varies linearly with plausibility.

### Modeling the link between plausibility and repetition

In the following, we formalize the simulation-based predictions by Fazio et al. (2019) as a precise mathematical model. The simulation is based on the idea that each statement is assigned a particular plausibility value ($p_i$), which determines the probability of “true” responses for that statement. However, while Fazio et al. (2019) approximated the predicted response probabilities by simulating a large number of hypothetical latent truth values ($y^*$), it is possible to directly model the marginal probability of judging a statement as true. In fact, simulating normally distributed values, which are then thresholded into binary responses, is equivalent to a simple probit model (Amemiya, 1981). The probability of categorizing a new statement as true is thus

3 $$\begin{aligned} \theta _i^\text {new} = P(y_i^\text {new} = \text {``true''}) = \Phi \left( \frac{p_i - 0.5}{\sigma }\right) . \end{aligned}$$

For repeated statements, the effect of fluency due to repetition ($f_i$) is incorporated by adding it to the baseline plausibility value,

4 $$\begin{aligned} \theta _i^\text {rep} = P(y_i^\text {rep} = \text {``true''}) = \Phi \left( \frac{p_i + f_i - 0.5}{\sigma }\right) . \end{aligned}$$

Rather unconventionally, the model does not assume a *standardized* normal distribution of latent values and a cutoff of zero for thresholding. Instead, Fazio et al. (2019) implicitly defined a link function with a standard deviation of $\sigma =0.5$ and a threshold of 0.5 to compute the predicted probabilities.^2^ The unusual threshold explains why the value 0.5 is subtracted in the two probit functions. Overall, the probit functions are theoretically equivalent to Equations (1) and (2).

**Fig. 1:**
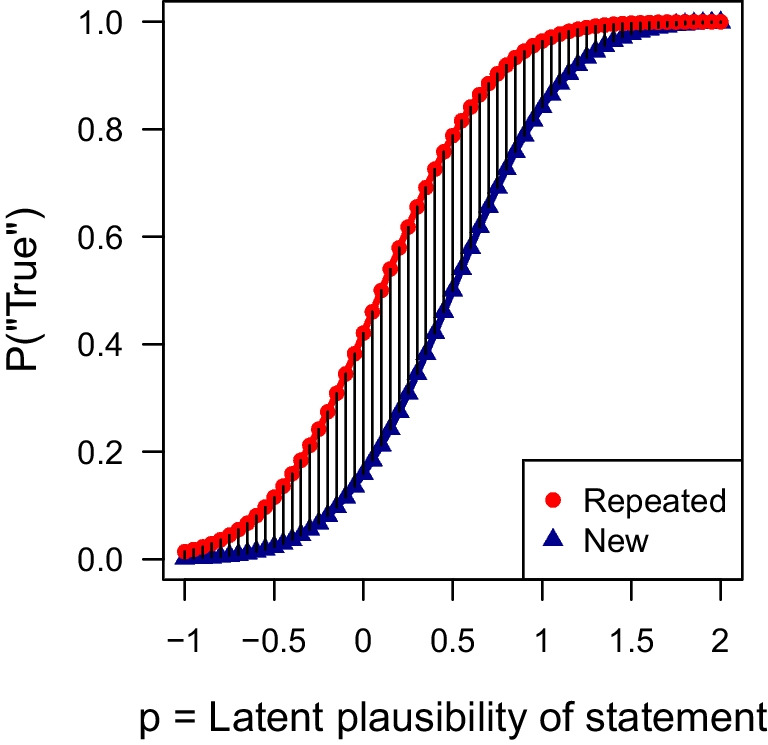
Transformation of a statement’s plausibility $p_i$ to the observed probability of giving a “true” response

Figure 1 illustrates the mapping of the plausibility of a statement onto the probability of judging a statement as true. The curve for new statements (in blue triangle shapes) represents the link between plausibility and the probability of judging a statement as true. The curve for repeated statements (in red round shapes) changes according to the assumption regarding the interaction of the baseline plausibility and the repetition effect. For the plot, we assume that the additive fluency effect $f_i = 0.4$ is constant for all statements on the latent *plausibility scale*. Crucially, assuming a constant, additive effect of repetition implies the absence of an interaction (for details, see below). Due to the nonlinear probit transformation, the effect of repetition on the *probability scale* (illustrated by vertical black lines) diminishes for highly implausible and plausible statements compared to ambiguous statements.

**Fig. 2:**
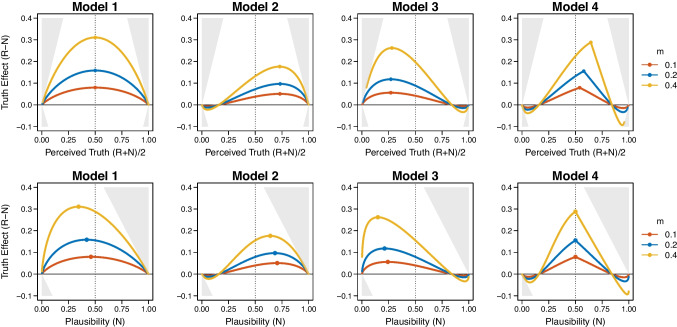
Predicted truth effect of the four models by Fazio et al. (2019). Upper row: The x-axis corresponds to average truth judgments across new (*N*) and repeated (*R*) statements. Lower row: Alternatively, the plausibility of statements on the x-axis can be operationalized by the average truth judgments for new (*N*) statements only. In all plots, gray areas indicate impossible data points

We now focus on the relationship between a statement’s baseline plausibility $p_i$ and the effect of fluency $f_i$ induced by repetition. The modeling framework allows specifying different assumptions on whether the size of the truth effect depends on the baseline plausibility or is constant. Also, one can study whether the predicted truth effect is largest for statements with low, medium, or high plausibility.

Fazio et al. (2019) proposed four different models: Model 1 assumes that repetition has a constant effect $f_i = m_1$ independent of the baseline plausibility $p_i$, which implies that the impact of fluency is identical across all statements. This corresponds to the absence of an interaction because the difference between repeated and new statements does not depend on plausibility. In contrast, Models 2 and 3 assume a specific type of interaction, namely, a linear dependence. Model 2 assumes a larger fluency effect for *plausible* statements, expressed as $f_i = m_2 p_i$, and Model 3 assumes a larger fluency effect for *implausible* statements, defined as $f_i = m_3 (1-p_i)$. Lastly, Model 4 implements a triangular function with a peak at $p_i = 0.5$ (scaled by the parameter $m_4$), thereby assuming that the largest fluency effect occurs for statements with medium plausibility. Jointly with the probit-link function, the models thus make the following predictions:

5 $$\begin{aligned} \theta ^\text {rep}_i \mid \mathcal {M}_1&= \Phi \left( \frac{p_i+m_1 - 0.5}{\sigma }\right) \end{aligned}$$

6 $$\begin{aligned} \theta ^\text {rep}_i \mid \mathcal {M}_2&= \Phi \left( \frac{p_i+m_2 p_i - 0.5}{\sigma }\right) \end{aligned}$$

7 $$\begin{aligned} \theta ^\text {rep}_i \mid \mathcal {M}_3&= \Phi \left( \frac{p_i+m_3(1-p_i) - 0.5}{\sigma }\right) \end{aligned}$$

8 $$\begin{aligned} \theta ^\text {rep}_i \mid \mathcal {M}_4&= \Phi \left( \frac{p_i+2 m_4(0.5-|p_i-0.5|) - 0.5}{\sigma }\right) \end{aligned}$$

The upper row of Fig. 2 illustrates the model predictions for the three parameter values $m = 0.1$, $m = 0.2$, and $m = 0.4$ used for the simulations by Fazio et al. (2019). The derived patterns correspond to the simulation-based predictions in the original paper, with the difference of being exact, analytical curves without any simulation noise.

### Operationalization of plausibility

The truth effect is commonly defined as the difference between the observed relative frequencies of “true” responses for repeated (*R*) and new (*N*) statements (i.e., $R-N$). As shown in the first row of Fig. 2, Fazio et al. (2019) plotted the truth effect against the perceived truth of statements, operationalized as $(R+N)/2$, the overall relative frequency of “true” responses averaged across new and repeated statements. This approach was motivated by a theoretical paper of Chapman and Chapman (1988) who highlighted conceptual issues of studies measuring lateral advantage. Chapman and Chapman (1988) focused on genuine and artificial relationships between a difference score (i.e., the difference of two proportions) and overall accuracy (i.e., the mean of two proportions). An artificial association of lateral advantage can emerge for two reasons: First, the maximum of the difference score is constrained by the average (e.g., if the average is 90%, the difference can maximally be 20%); and second, differences in the variances of the two proportions can produce a curvilinear relationship.^3^ Fazio et al. (2019) adopted this reasoning to investigate the relationship between plausibility and the size of the truth effect. For this purpose, they also conceptualized the two variables as the average proportion, $(R+N)/2$, and the difference in proportions, $R-N$.

We agree that the psychometric issues highlighted by Chapman and Chapman (1988) also apply to the case of (binary) truth judgments and can explain the curvilinear relationship between the difference score and the average. However, Fazio et al. ’s (2019) approach of defining and measuring plausibility as the mean across new and repeated statements raises concerns. Such a definition gives the impression as if the size of the repetition effect depended both on the proportion *N* of “true” responses *before* the manipulation and on the proportion *R* *after* the manipulation. Essentially, the operationalization $(R+N)/2$ reflects not only the baseline plausibility but also the repetition effect itself, which is problematic from a theoretical perspective.

Conceptually, it is preferable to consider the initial perceived accuracy of statements as the operationalization of the baseline plausibility. This better aligns with the model’s assumption that each statement possesses a baseline plausibility $p_i$ which increases to $p_i+f_i$ due to repetition. The panels in the lower row of Fig. 2 plot the predicted truth effect as a function of the relative frequency of “true” responses for new statements only (i.e., *N*). This conceptually preferable representation implies that the truth effect will necessarily be larger for implausible statements than for plausible statements due to ceiling effects (i.e., the probability of judging a statement as true cannot increase above one as indicated by gray areas for impossible data points).

For the data of Fazio et al. (2019), the values of the two alternative operationalizations *N* and $(R+N)/2$ are highly correlated across items ($\rho = 0.993$, 95% CI: [0.989, 0.996]) and have very small mean absolute deviations of 0.21. Despite the high overlap of these two variables, the operationalization of perceived truth in terms of new statements only (*N*) avoids the reliance on post-manipulation values and provides a clearer specification of the relationship between plausibility and the truth effect (Bringmann et al., 2022).

## Assumptions about scale transformations

Fazio et al. (2019) assert that “repetition increases participants’ internal representation of truth equally for all statements” (p. 1710). This argument implies the absence of an interaction, meaning that plausibility does not moderate the effect of fluency on truth judgments. Put differently, the effect of fluency on the truth effect is assumed to be constant across different levels of plausibility. However, it is crucial to scrutinize the robustness of such conclusions about interactions and additive effects in the context of the underlying model assumptions.

To interpret the interaction, it is essential to critically evaluate the chosen link function, which nonlinearly transforms the scale of the perceived accuracy of the statements (see Fig. 1). In the following, we examine the importance of this assumption for interpreting whether the effects of repetition and plausibility are additive. The essence of the matter lies in making auxiliary assumptions explicit and discerning which aspects of the model are testable and which are not. To explain the consequences of scale transformations, we focus on Model 1 which assumes a constant fluency effect.

### Additivity and interpretable interactions

The interpretation of interactions in statistical models depends on the scale of measurement and the application of nonlinear transformations (Loftus, 1978). The scale of measurement, delineating the nature of variables, plays a crucial role since the effect of one variable on another may differ across different scales (Stevens, 1946). In our case, the probability scale for binary responses, which is bounded between zero and one, is nonlinearly transformed to an unbounded latent scale by a link function in the model. Nonlinear transformations such as the logarithm, the exponential function, or the probit transformation can affect the interpretation of interactions (Box & Cox, 1964). The application of nonlinear transformations can introduce curvature or changes in the relationship between variables, thus influencing the nature of interaction effects (Fox, 2015). Some interactions can even be removed by nonlinear transformations, meaning that two factors that are additive on one scale may exhibit an interaction on a different scale (Loftus, 1978; Wagenmakers et al., 2012).

**Fig. 3:**
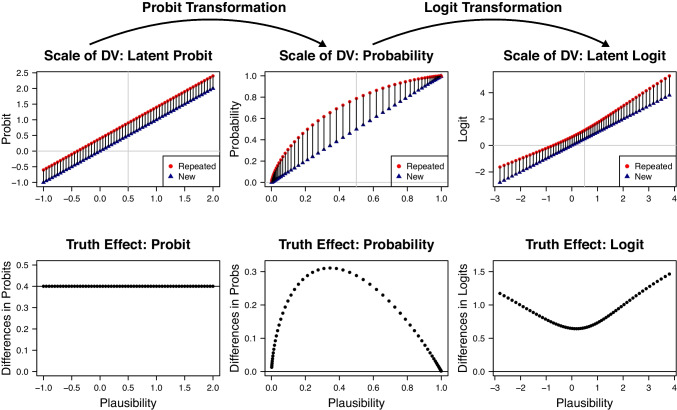
Comparison of the interaction between truth effect and plausibility on different scales. The arrows on top of the figure illustrate the application of the probit and logit-link functions, respectively. In the upper row, vertical segments represent the truth effect. In the interaction plots in the lower row, the differences between repeated and new statements are shown as a function of plausibility

The modeling approach by Fazio et al. (2019) assumes that the probability of new statements being judged as true depends on the latent plausibility $p_i$, with values ranging from $-1$ to 2 on a probit scale. According to Model 1, a constant fluency effect $f_i=m_1$ is added to the plausibility values for repeated statements. For instance, assuming a fluency effect of 0.4, the predictions for repeated statements being judged as true are computed as $p_i + 0.4$, with values spanning from $-0.6$ to 2.4. On the latent probit scale, the constant difference between new and repeated statements across the whole probit scale is illustrated in the first column of Fig. 3. However, the formal model makes predictions about perceived accuracy and the effect of repetition on the probability scale. Consequently, when applying the probit-link function to a range of values for new and repeated statements, their difference is no longer constant. As illustrated in the middle column of Fig. 3, applying the probit-link function introduces variability in these differences, meaning that the size of the truth effect depends on plausibility.

The reason why we see such a pattern is that the probit-link function maps unbounded values onto a bounded probability range between zero and one, and such a mapping is not linear. Values approaching negative or positive infinity are getting closer to the floor and ceiling of the bounded probability range, respectively. As a side effect, differences between two probit values close to zero (e.g., 1.00 and 0.60) result in larger differences on the probability scale than equally sized differences between two large probit values (e.g., 2.14 and 1.74).^4^ Importantly, differences between the transformed values also depend on the specific type of transformation applied. For instance, when applying the logit transformation, differences between values close to zero become smaller compared to those obtained with the probit transformation. In contrast, for values at the extremes of the logit scale, differences are larger than those produced by the probit transformation.^5^

In the modeling approach by Fazio et al. (2019), the link function and the scale determine whether plausibility and repetition have merely additive effects or show an interaction. The model assumes a specific link function between the latent construct and the observed quantities, namely, a probit transformation (see Equations (3) and (4)). Any conclusions drawn on the model depend on this link function and the corresponding scale. Essentially, the truth effect refers to the difference in the probability of a statement being perceived as true when it is repeated compared to when it is presented for the first time. The primary focus revolves around examining whether the size of this difference is moderated by the baseline plausibility.

The graphical representation in Fig. 3 presents interaction plots to facilitate a comprehensive understanding of how interactions manifest across different scales and transformations (Cleveland, 1993). The left column corresponds to Model 1 and assumes that fluency has an additive constant effect on the latent probit scale. Transforming these values to probabilities (middle column) and applying a logit instead of a probit transformation (third column) result in a completely different interaction pattern, characterized by a larger repetition effect for very low and very high plausibility. Overall, Figure 3 thus illustrates that applying different link functions to the response probabilities changes the presence and the nature of the interaction between plausibility and repetition.

## Statistical model selection

Fazio et al. (2019) derived simulation-based predictions through precise equations, which can be implemented in any statistical software. However, the question remains on how to best test the model using empirical data. In an online study, Fazio et al. (2019) presented participants with statements from a large range of plausibility. After being exposed to half of the statements, participants had to judge the truth of new and repeated statements. Separately for each statement, the proportion of “true” responses was computed in the new and the repeated condition (referred to as *N* and *R*, respectively). To facilitate a comparison with the predictions of the computational models, Fazio et al. (2019) plotted the truth effect, defined as $R-N$, against the perceived truth, operationalized as $(R+N)/2$. The goal was to test whether observed judgments were in line with the hypothesis that the latent fluency effect due to repetition is constant. For this purpose, the authors focused on a crucial prediction of Model 1, namely, that the peak of the fitted curve (an inverted U-shape) is located at a perceived accuracy of $(R+N)/2 =.50$ (cf. Figure 2). This null hypothesis was statistically tested by conducting a quadratic regression analysis and performing a tailored bootstrapping procedure. The test was not significant, leading Fazio et al. (2019) to conclude that Model 1 provides an adequate description of the data.

The testing approach of Fazio et al. (2019) is valid but has some limitations that do not allow us to draw strong conclusions. First, the non-significant test result cannot be interpreted as evidence for the null hypothesis without considering the statistical power of the test. Their analysis relied only on 80 statements. Given that the analysis focuses on a property of the quadratic regression that is difficult to estimate (i.e., the peak of the inverted U-curve), the limited sample size presumably results in relatively low statistical power. To evaluate the relevance of this possible issue, we performed a power analysis by generating 1,000 datasets for each model with *m* values of 0.1, 0.2, and 0.4. Quadratic regression was then applied to these datasets, followed by the construction of 5,000 bootstrap samples obtained by resampling 80 statements in the simulated datasets with replacement. Implementing the approach by Fazio et al. (2019), we calculated the proportion of bootstrap samples whose 95% confidence intervals excluded the midpoint .50. Simulations showed that, when datasets were simulated by Model 1, rejection rates were $5.5\%$ for $m = 0.1$, $6.7\%$ for $m = 0.2$, and $9.6\%$ for $m = 0.4$. Note that these values exceed the nominal significance level of $\alpha =5\%$. For the datasets generated by Models 2 and 3, all 95% bootstrapped confidence intervals excluded the midpoint .50, thus achieving a very high statistical power close to 100% for all *m* values. However, the power for Model 4 was notably low for small *m* values, with rejection rates of $11.6\%$ for $m = 0.1$ and $39.2\%$ for $m = 0.2$. Only for $m = 0.4$, the 95% bootstrapped confidence intervals rejected the midpoint 100% of the time. These results suggest that, especially for low values of *m*, distinguishing whether Model 1 or 4 generated the data using quadratic regression is challenging.

Second, it was not directly assessed whether the complete functional shapes predicted by the four models can describe the observed patterns in the data. Instead, only a single property of one of the four models was tested. In fact, the quadratic curve fitted to the data necessarily differs from the complex and nonlinear curves predicted by all of the four models. Fitting a simple quadratic regression overlooks the theoretically grounded, model-specific nonlinearities and interactions between the parameters that are essential for accurately capturing the functional shapes predicted by the four models. This potentially results in an incomplete understanding of the relationship between plausibility and the truth effect. For instance, quadratic regression cannot capture the triangular shape of Model 4 and predicts a shape closer to that of Model 1, which may lead to biased inferences.

The original testing approach focused on the question of whether the mode of the quadratic, inverted U-shape is at a midpoint plausibility value of .50 as predicted by Model 1. In discussing the predictions of the four models, Fazio et al. (2019) state that “when the repetition effect was larger for implausible statements [Model 3], the midpoint was below .50, and when the repetition effect increased with plausibility [Model 2] or was largest for items in the middle of the plausibility scale [Model 4], the midpoint was above .50” (p. 1709). Given that a symmetric pattern around .50 was observed, and that this pattern visually resembles the predictions of Model 1, Fazio et al. (2019) concluded that the effect of repetition is constant regardless of plausibility. However, other models make similar predictions. For instance, Model 4 also predicts an approximately symmetric shape with a mode at .55 (for $m_4 = 0.1$), which is inside the reported $95\%$ confidence interval of [.49, .59]. This renders Model 4 compatible with the experimental results as well. Since the models were not directly compared against each other, the statistical analyses thus provide only weak evidence for Model 1, while remaining silent on the other three models.

### Bayesian modeling

A solution for the statistical issues mentioned above is to fit all four models to the empirical data. This allows for assessing parameter estimates, functional shapes, and statistical model selection. Bayesian model comparison relies on the Bayes factor, which balances the goodness of fit against the complexity of models (Kass & Raftery, 1995; Heck et al., 2023). In the present case, all four models under consideration have the same number of parameters but predict distinct functional forms (i.e., the parameter *m*). This poses an issue for commonly used information indices, such as the Akaike or Bayesian information criteria, since they merely rely on the number of parameters as a proxy for model complexity. In contrast, the Bayes factor is particularly informative and suitable for the present scenario because it takes the varying complexity of different functional shapes into account (Myung, 2000). Also, by defining informed prior distributions for the parameters, one can ensure that models actually reflect substantive and theoretical assumptions (Lee & Vanpaemel, 2018).

We implemented the simulation-based predictions of Fazio et al. (2019) as a Bayesian model, which can be fitted to empirical data. Each statement *i* judged by participant *j* has a plausibility parameter $p_i$ which is modeled by a random-effects distribution across statements. More precisely, $p_i$ follows a normal distribution with mean $\mu ^p$ and standard deviation $\sigma ^{ p\!}$. As a hyper-prior for the average plausibility $\mu ^p$, we assume a normal distribution centered at 0.5 with a standard deviation of 1. This prior implies a uniform distribution at the probability scale because the threshold value of 0.5 is subtracted from the mean at the probit scale. For the hyper-prior $\sigma ^{ p\!}$, we assume a positive truncated normal distribution with mean 0 and standard deviation 1. The effect of repetition (i.e., the increase in plausibility due to fluency *f*) is modeled with the parameter *m* in each model. Since it is likely that many properties of statements moderate the size of the truth effect, we assume level-2 residuals for the predicted probability of judging repeated statements as true. More precisely, we added random effects for the item-specific truth effect of each statement by including the $v_i$ parameter as an additive term in the numerator of Equations (5) to (8).

Fitting the models to individual-level as opposed to aggregated data offers the benefit of yielding more accurate estimates by preserving individual differences. Moreover, it reduces the risk of misleading inferences that can arise from fitting nonlinear models to aggregated data (Estes, 1956; Estes & Maddox, 2005). Furthermore, maximally-defined participants and items crossed-random effects also reduce the Type I error rate (Barr et al., 2013). To account for the fact that individual differences among participants also affect the probability of judging statements as true, we added random effects for participants.

More precisely, we added the parameter $u_{j}$ which can be understood as response bias. Larger $u_{j}$ values mean that participant *j* generally provides more “true” responses to the presented statements. Both random effects $v_i$ and $u_{j}$ follow a normal distribution with mean 0 and standard deviations $\sigma ^{v}$ and $\sigma ^{u}$, respectively. As a hyper-prior for the standard deviations $\sigma ^{v}$ and $\sigma ^{u}$, we again assume a positive truncated normal distribution with mean 0 and standard deviation 1.

Our choice of priors reflects theoretical considerations by ensuring prior-predictive distributions that match typical data on the truth effect (Lee & Vanpaemel, 2018). We set $\sigma$, the standard deviation of latent probit values, to a fixed value of 0.5 (Fazio et al., 2019). This constant determines the slope of the link function in Equations (5) to (8). Fazio et al. (2019) adopted the values 0.1, 0.2, and 0.4 for the parameter *m*, which determines the magnitude of the repetition effect as illustrated in Fig. 2.

Since the four models differ in their functional shapes, the same value of *m* results in a different average repetition effect in each model version. Hence, defining the same prior distribution for the parameter *m* in each model is potentially problematic. As a remedy, it is necessary to scale the prior for *m* such that the implied size of the truth effect is approximately equal for all four models. For this purpose, we generated datasets for each model with *m* values ranging from 0.01 to 0.50 (with increments of 0.01) and then computed the average truth effect for each combination of model and *m* value. We searched for those *m* values for each model that provided approximately the same average truth effect as observed in Fazio et al. (2019), which was .04. This scaling procedure resulted in matched *m* values of 0.11, 0.27, 0.19, and 0.38 for Models 1 to 4, respectively. These values were then used as the means of the positive truncated normal distributions defined as priors for *m* for each model. Each prior has a standard deviation of 0.1, which assigns substantial weight to values in the range from 0.1 to 0.4 for each model.^6^

The likelihood function depends on the predicted probabilities of giving a “true” response for new and repeated statements as defined in Equations (5) to (8). These probabilities $\theta$ are used as parameters for a Bernoulli distribution of the binary truth judgment $y_{ij}$ of whether statement *i* was judged as true by participant *j*. The term $\mathcal {M}_k$ in (10) corresponds to the specific model variant *k* whereby the predictions for repeated statements differ depending on the four model variants,

9 $$\begin{aligned} y^\text {new}_{ij}&\sim \text {Bernoulli}(\theta ^\text {new}_{ij})\end{aligned}$$

10 $$\begin{aligned} y^\text {rep}_{ij}&\sim \text {Bernoulli}(\theta ^\text {rep}_{ij} \mid \mathcal {M}_k). \end{aligned}$$

**Fig. 4:**
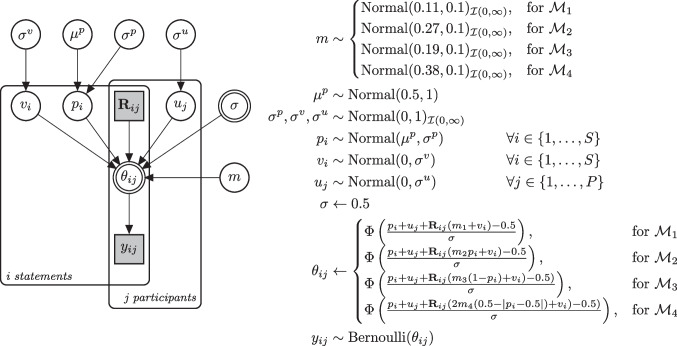
Graphical representation of the Bayesian model. *S* and *P* are the numbers of statements and participants, respectively. The data vector $\textbf{R}$ is an indicator variable with values 1 and 0, denoting whether statement *i* judged by participant *j* was repeated or new, respectively. For new statements, the effect of repetition is eliminated since $\textbf{R}_{ij} = 0$

### Implementation of the model

The four Bayesian models were implemented using the probabilistic programming language Stan (Stan Development Team, 2024b). Figure 4 provides an overview of all parameters, prior distributions, and the likelihood function of the model. We estimated the posterior distribution of the parameters by fitting the model to data using Hamiltonian Monte Carlo sampling in R (R Core Team, 2023) via the *rstan* package (Stan Development Team, 2024a). For parameter estimation, we fitted the four models using eight chains, each with 1,000 warm-up iterations and 10,000 posterior samples after warm-up.

Convergence was assessed both visually and using common convergence diagnostics. The chains mixed well for all parameters, as indicated by trace plots. The effective sample size (ESS) of the posterior samples revealed that the *m* parameters achieved a sufficient precision (all $\text {ESS}_m>$ 67,000), indicating small autocorrelation. Moreover, all $\hat{R}$ values were below 1.02. Overall, this supports the use of the posterior samples for reliable inferences (Vehtari et al., 2021; Gelman & Rubin, 1992).

To compute Bayes factors for model selection, we employed the bridge sampling method (Bennett, 1976; Meng & Wong, 1996) using the *bridgesampling* package (Gronau et al., 2020). All data and analysis code are publicly available on the Open Science Framework repository, accessible at https://osf.io/q48jv/.

### Reanalysis of two datasets

**Fig. 5:**
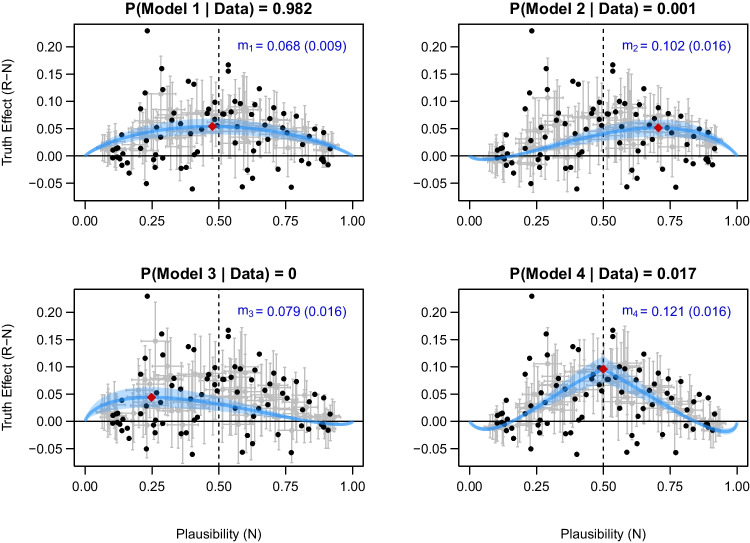
Fit of the four competing models for the data of Fazio et al. (2019). Black dots represent the statement-specific observed truth effect. In the upper right corner of each panel, the posterior mean and SD of the parameter *m* are shown in blue color. Light blue bands correspond to $95\%$ credible intervals. Statement-specific posterior predictions are shown with 95% credible intervals by gray crosses. Red diamonds indicate the peak truth effect for each model

Fitting the Bayesian models to the data of Fazio et al. (2019) resulted in the following estimates of the parameter *m* in the four models: $m_1 = 0.068$ (posterior $\text {SD} = 0.009$), $m_2 = 0.102$ (posterior $\text {SD} = 0.016$), $m_3 = 0.079$ (posterior $\text {SD} = 0.016$) and $m_4 = 0.121$ (posterior $\text {SD} = 0.016$). These results suggest that, for all four models, the posterior estimates of the parameter *m* were mostly smaller than the values assumed in the original simulation (i.e., 0.1, 0.2, and 0.4). As mentioned above, the interpretation of the parameter *m* is not identical across the four models due to the different assumptions. The same value of *m* results in different predictions of the size of the truth effect (see Equations (5) to (8)). Due to the nonlinear probit transformation, it is difficult to interpret the size of the truth effect based only on the posterior estimates of the parameter *m*. To facilitate the interpretation of the repetition effect, we examined the fitted curves for each model. Figure 5 shows that, on the probability scale, the truth effect is descriptively larger for ambiguous statements than for highly plausible and implausible statements. Based on a visual inspection, it is difficult to say which of the models fits the data best.

We used Bayes factors for statistical model selection. The Bayes factors indicated strong evidence for Model 1 against Model 4 ($\text {BF}_{14} = 56.42$). Furthermore, Model 1 clearly outperformed both Model 2 ($\text {BF}_{12} > 10^3$) and Model 3 ($\text {BF}_{13} > 10^4$). Assuming equal model prior probabilities, Model 1 had the highest posterior probability of 0.982. This result provides strong evidence for the assumption that the additive effect of fluency on the probit scale is constant for ambiguous, plausible, and implausible statements, thus supporting the conclusions of Fazio et al. (2019).

**Fig. 6:**
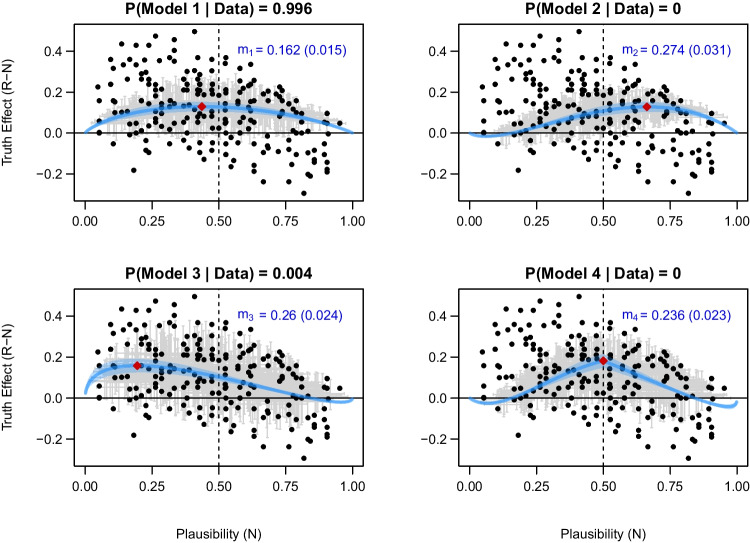
Fit of the four competing models for the data of Nadarevic et al. (2018) Experiment 2. Black dots represent the statement-specific observed truth effect. In the upper right corner of each panel, the posterior mean and SD of the parameter *m* are shown in blue color. Light blue bands correspond to $95\%$ credible intervals. Statement-specific posterior predictions are shown with 95% credible intervals by gray crosses. Red diamonds indicate the peak truth effect for each model

We also fitted the four Bayesian models to another dataset, namely, Experiment 2 of Nadarevic et al. (2018). Thereby, we aimed to assess the models’ validity and predictive power using an independent dataset. We chose this dataset since it had a sufficiently large variance in the plausibility of the statements presented in the experiment. The estimated sizes of the truth effect in terms of the parameter *m* for each model were $m_1 = 0.162$ (posterior $\text {SD} = 0.015$), $m_2 = 0.274$ (posterior $\text {SD} = 0.031$), $m_3 = 0.260$ (posterior $\text {SD} = 0.024$) and $m_4 = 0.236$ (posterior $\text {SD} = 0.023$). The posterior estimates for *m* were generally larger than those for the data by Fazio et al. (2019), and closer to the values assumed for the original simulations. The larger effect size of the truth effect is also reflected in Fig. 6 by the fitted curves for the four models, which span a larger range on the *y*-axis. However, it is again difficult to compare the goodness of fit of the four models based on a visual inspection.

The model comparison using Bayes factors provided strong evidence in favor of Model 1 over Model 2 ($\text {BF}_{12} > 10^5$), Model 3 ($\text {BF}_{13} > 10^2$), and Model 4 ($\text {BF}_{14} > 10^3$). Assuming equal prior probabilities, Model 1 was clearly the most probable model given the data with a posterior probability of 0.996. This provides evidence for the assumption that the repetition-induced truth effect is constant on the probit scale. Thereby, the results align with the conclusions of Fazio et al. (2019) and our reanalysis of their data.

To check the robustness of the model-selection results, we also employed leave-one-out cross-validation (LOOCV; Vehtari et al., 2017) using the *loo* package (Vehtari et al., 2024). This comparison was only possible for the data of Nadarevic et al. (2018). Due to computational limitations, we could not obtain results for the data of Fazio et al. (2019) as the log-likelihood matrices were too large to process. The results of LOOCV were in line with the model selection based on Bayes factors. The differences in predictive accuracy of the models, as indexed by Expected Log Predictive Density (ELPD), suggested that Model 1 was the best model (ELPD$$ = -5,409.12$$, LOOIC$$ = 10,818.2$$), followed by Model 3 (with a difference in ELPD$$ = -6.85$$, $$\text {SE}_{\text {diff}} = 4.78$$, LOOIC$$ = 10,831.9$$), Model 4 (with a difference in ELPD$$ = -8.50$$, $$\text {SE}_{\text {diff}} = 4.85$$, LOOIC$$ = 10,835.2$$) and Model 2 (with a difference in ELPD$$ = -13.89$$, $$\text {SE}_{\text {diff}} = 4.53$$, LOOIC$$ = 10,846.0$$).

### Relevance of random effects for participants

Our models were fitted to trial-level data to circumvent aggregation artifacts (Estes, 1956). Thus, besides random effects for statements, we also included random effects for participants. To assess the relevance of these participant-level random effects, we checked their variances. Large variances indicate substantial variation among participants’ judgments.

In the reanalysis of the data by Fazio et al. (2019), the estimated variance of the participant-level intercept parameter $u_{j}$ was 0.076, with a 95% highest density interval (HDI) of [0.071, 0.082], excluding zero. In the reanalysis of the data by Nadarevic et al. (2018), the estimated variance of participant-level intercept parameters $u_{j}$ was smaller ($\text {Mean}_{\text {var}} = 0.01$), but the 95% HDI, [0.007, 0.014], still excluded zero. These results suggest that adding participant-level random effects helped in capturing variation between participants.

### Sensitivity analyses

In Bayesian analysis, models with different prior distributions often yield a comparable fit to the data. Especially for Bayes factors, sensitivity analysis becomes imperative (Heck et al., 2023; Gelman et al., 1995; Berger, 1990). The central question is whether and how much posterior inferences change when models with different prior specifications are employed. It is possible that while the present models adequately fit the data, posterior inferences may differ under sensible alternative priors (Weiss, 1996; Canavos, 1975). Sensitivity analysis thus serves as a crucial technique for comprehending and quantifying the impact of different priors on the results (Borgonovo, 2023). Specifically, sensitivity analysis facilitates the assessment of robustness and reliability, and enables the identification of influential parameters, potential sources of uncertainty, and aspects where the model may be particularly sensitive to changes in priors or data (Gelman et al., 1995; Skene et al., 1986; Berger, 1990).

#### Model-invariant priors for *m*

Modeling the repetition effect in two independent datasets provided consistent evidence for Model 1. Notably, the four competing models only differ with respect to the predictions for repeated statements, which are determined by the parameter *m*. Hence, the prior distribution for *m* is crucial, as it determines the extent to which the prior predictions of the four models align with the data.

**Fig. 7:**
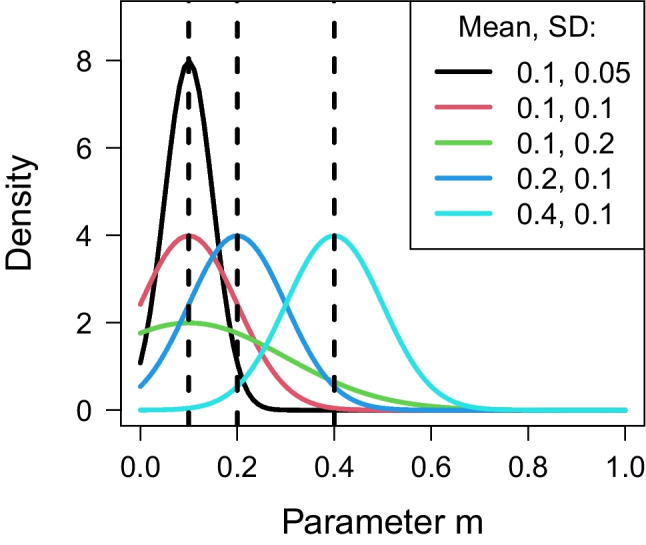
Truncated normal distributions used as model-invariant priors for the parameter *m* in the sensitivity analysis. Dashed vertical lines represent the values assumed by Fazio et al. (2019)

**Table 1:** Results of the sensitivity analysis of model-invariant priors for the parameter *m*

	$\text {BF}_{12}$	$\text {BF}_{13}$	$\text {BF}_{14}$	$P(\mathcal {M}_1)$	$P(\mathcal {M}_2)$	$P(\mathcal {M}_3)$	$P(\mathcal {M}_4)$
**Prior for** $\varvec{m}$	Experiment of Fazio et al. (2019)						
$\text {Normal}(0.1,0.05)_{\mathcal {I}(0,\infty )}$	$>10^2$	$>10^4$	1.66	**0.622**	0.003	0.000	0.375
$\text {Normal}(0.1,0.1)_{\mathcal {I}(0,\infty )}$	$>10^2$	$>10^4$	1.73	**0.632**	0.003	0.000	0.366
$\text {Normal}(0.1,0.2)_{\mathcal {I}(0,\infty )}$	$>10^2$	$>10^4$	1.81	**0.642**	0.003	0.000	0.355
$\text {Normal}(0.2,0.1)_{\mathcal {I}(0,\infty )}$	$>10^2$	$>10^4$	1.08	**0.518**	0.003	0.000	0.479
$\text {Normal}(0.4,0.1)_{\mathcal {I}(0,\infty )}$	86.22	$>10^4$	0.44	0.302	0.004	0.000	**0.694**
	Experiment 2 of Nadarevic et al. (2018)						
$\text {Normal}(0.1,0.05)_{\mathcal {I}(0,\infty )}$	$>10^6$	$>10^3$	$>10^4$	**1.000**	0.000	0.000	0.000
$\text {Normal}(0.1,0.1)_{\mathcal {I}(0,\infty )}$	$>10^6$	$>10^2$	$>10^3$	**0.998**	0.000	0.002	0.000
$\text {Normal}(0.1,0.2)_{\mathcal {I}(0,\infty )}$	$>10^5$	$>10^2$	$>10^3$	**0.996**	0.000	0.003	0.000
$\text {Normal}(0.2,0.1)_{\mathcal {I}(0,\infty )}$	$>10^5$	$>10^2$	$>10^3$	**0.996**	0.000	0.004	0.000
$\text {Normal}(0.4,0.1)_{\mathcal {I}(0,\infty )}$	$>10^4$	34.76	$>10^2$	**0.971**	0.000	0.028	0.001

The original priors utilized in our Bayesian model were scaled for each model variant. This scaling procedure ensured that the chosen priors imply the same average truth effect. However, it is not clear whether it is necessary to employ this scaling procedure and how sensitive the results are to non-scaled, fixed priors. To answer these questions, we ran a sensitivity analysis with the same priors for all models. As alternative priors, we used positive truncated normal distributions with varying hyper-parameters $\mu$ and $\sigma$: $\text {Normal}(0.1,0.05)_{\mathcal {I}(0,\infty )}$, $\text {Normal}(0.1,0.1)_{\mathcal {I}(0,\infty )}$, $\text {Normal}(0.1,\!0.2)_{\mathcal {I}(0,\infty )}$, $\text {Normal}(0.2,\!0.1)_{\mathcal {I}(0,\infty )}$, and $\text {Normal}$ $(0.4,0.1)_{\mathcal {I}(0,\infty )}$. Similar to the original priors, these priors also assign substantive weight to values and deliberately favor truth effect values spanning from 0.1 to 0.4. Figure 7 illustrates these priors.

For each prior, we performed Bayesian model fitting, and obtained Bayes factors and posterior probabilities of the four models. Table 1 summarizes Bayes factors and posterior model probabilities under the fixed alternative priors. The sensitivity analysis unveiled notable differences in the models fitted to the data of Fazio et al. (2019). With the exception of the prior distribution $\text {Normal}(0.4,0.1)_{\mathcal {I}(0,\infty )}$, Model 1 consistently exhibited the highest posterior probability among the four models across all alternative priors. However, an examination of the Bayes factors indicated only ambiguous evidence favoring Model 1 over Model 4. In fact, under the prior $\text {Normal}(0.4,0.1)_{\mathcal {I}(0,\infty )}$, Model 4 demonstrated a higher posterior probability than Model 1. Similarly, the Bayes factors under this prior provided no evidence favoring one model over the other. These findings emphasize the variability in model performance depending on the choice of prior. A prior that performs well for one model may cause another to underperform, highlighting the need for careful prior specification. Overall, the results suggest that the sensitivity of model comparisons to prior choices necessitates scaling the priors to achieve more consistent and robust conclusions.

The sensitivity analysis for the data of Nadarevic et al. (2018) produced Bayes factors and posterior probabilities similar to those obtained using the original priors. This finding indicates that our modeling approach exhibits lower sensitivity to variations in the prior for the parameter *m* when applied to the data of Nadarevic et al. (2018). The mixed sensitivity results across datasets indicate that the outcomes of the analyses depend on the specific dataset to which the models are fitted. Consequently, to ensure robust conclusions, priors should be carefully scaled to maintain consistency in inference patterns across reanalyses of different datasets.

**Fig. 8:**
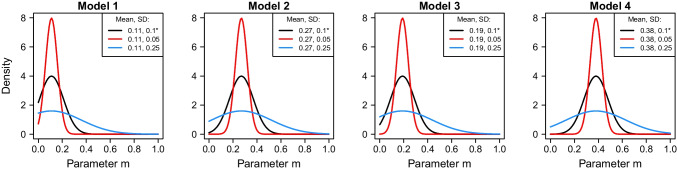
Truncated normal distributions used in the sensitivity analyses for the model-specific priors of parameter *m*. *Priors used for the main analysis

**Table 2:** Results of the sensitivity analysis for the informativeness of model-specific priors for the parameter *m*

	$\text {BF}_{12}$	$\text {BF}_{13}$	$\text {BF}_{14}$	$P(\mathcal {M}_1)$	$P(\mathcal {M}_2)$	$P(\mathcal {M}_3)$	$P(\mathcal {M}_4)$
$\varvec{\sigma }$ **for** $\varvec{m}$ **prior**	Experiment of Fazio et al. (2019)						
$\sigma = 0.1^*$	$>10^3$	$>10^4$	66.15	**0.984**	0.001	0.000	0.015
$\sigma = 0.05$	$>10^3$	$>10^5$	$>10^5$	**1.000**	0.000	0.000	0.000
$\sigma = 0.25$	$>10^2$	$>10^4$	4.32	**0.811**	0.002	0.000	0.187
	Experiment 2 of Nadarevic et al. (2018)						
$\sigma = 0.1^*$	$>10^5$	$>10^2$	$>10^3$	**0.997**	0.000	0.003	0.000
$\sigma = 0.05$	$>10^5$	$>10^2$	$>10^4$	**0.997**	0.000	0.003	0.000
$\sigma = 0.25$	$>10^5$	$>10^2$	$>10^3$	**0.996**	0.000	0.004	0.000

*Note*. *Priors used for the main analysis. Scaled, model-specific priors for *m* were used with a mean parameter $\mu$ of 0.11, 0.27, 0.19, and 0.38 for Models 1 to 4, respectively

#### Informativeness of model-specific priors for *m*

The standard deviation of the prior for *m* is relatively small, making it rather restrictive. As a result, values are sampled from a constrained, informative range, which may influence the results. To address this potential limitation, we conducted an additional sensitivity analysis using two alternative priors: an overly restrictive (informative) prior ($\sigma = 0.05$) and a more relaxed (weakly informative) prior ($\sigma = 0.25$). These were compared with the original prior ($\sigma = 0.1$). All alternative priors followed a positive truncated normal distribution, with the means scaled specifically for each model (see Bayesian Modeling section above). Figure 8 illustrates the density functions of these priors, providing a visual representation of their differences.

For each prior, we performed Bayesian model fitting to compute Bayes factors and posterior probabilities for the four competing models. Table 2 summarizes the Bayes factors and posterior model probabilities obtained under both the original and alternative priors. The sensitivity analysis for the data by Fazio et al. (2019) revealed minimal variability in Bayes factors and posterior probabilities across different priors. Model 1 consistently emerged as the most probable model, particularly when compared to Model 4. The use of an overly restrictive prior ($\sigma = 0.05$) further strengthened the preference for Model 1. In contrast, increasing the standard deviation of the prior to ($\sigma = 0.25$), making it weakly informative, led to a slight decrease in the posterior probability of Model 1 to 0.811, while the posterior probability of Model 4 increased to approximately 0.187. Despite these changes, the Bayes factors continued to provide moderate evidence for Model 1 against Model 4 ($\text {BF}_{14} = 4.32$).

The sensitivity analysis for the data by Nadarevic et al. (2018) also yielded consistent results regardless of the restrictiveness of the prior for the parameter *m*. Across all standard deviations of the priors, Model 1 consistently emerged as the most favorable model compared to the remaining three models. These findings suggest that our modeling approach is robust against variations in prior specification for *m* when comparing restricted and relaxed priors.

## Discussion

We developed a Bayesian model of the repetition-based truth effect, which formalizes the simulation-based predictions of Fazio et al. (2019). Our model precisely specifies the hypothesized structure underlying the interaction between a statement’s plausibility and the effect of repetition. In plotting model predictions and data, we operationalized plausibility as the probability of providing “true” responses for new statements only (instead of averaging across both new and repeated statements). This provides a conceptually more valid representation of the link between plausibility and the truth effect while mitigating potential confounding factors associated with post-manipulation values. We showed that, by generating a large number of latent, normally distributed plausibility values, the simulation-based model by Fazio et al. (2019) essentially assumes a probit-link function, which determines the predicted probability of a statement being judged as true. The non-linearity inherent in the probit-link function has to be considered as a core assumption of the model since relying on other nonlinear link functions can result in different conclusions regarding the presence or absence of an interaction (Loftus, 1978).

Fazio et al. (2019) formulated the four competing models through mathematical equations and derived predictions using simulations. However, to compare the four models empirically, they provided only a relatively weak statistical test of a certain property of one model (i.e., about the location of the mode of the inverted U-curve). To improve the original analysis, we implemented the models using Bayesian hierarchical modeling. We fitted all four competing models to the data of Fazio et al. (2019) and to those of an independent group of researchers (Nadarevic et al., 2018). The possibility to fit the same model to multiple datasets highlights an advantage of formal models over simulation-based predictions since it allows us to test the robustness of the results (Guest & Martin, 2021; Smaldino, 2017). Moreover, by fitting the models to data, the complete shape of the predicted patterns is assessed instead of focusing only on a single aspect, such as the location of the mode.

Bayesian estimation showed that the truth effect, which is estimated by the parameter *m*, was larger in the experiment by Nadarevic et al. (2018) than in the original study by Fazio et al. (2019). Besides estimating model parameters and plotting fitted curves, we mainly focused on model comparison by means of posterior model probabilities. Fazio et al. (2019) interpreted the results of the original, indirect statistical test and their visual assessment as evidence for Model 1 and concluded that the truth effect is constant on the probit scale.

In a similar vein, across both datasets, Bayesian model comparison revealed clear evidence for Model 1 against the other three models, particularly Model 4. This outcome aligns with Fazio et al. ’s (2019) conclusion that there is no interaction between repetition and plausibility, such that the effect of fluency on plausibility is constant on the *probit* scale. However, when assessing the fitted curves of Models 1 and 4 on the *probability* scale (as plotted in Figs. 5 and 6), a consistent observation is that the most pronounced truth effect occurred for statements with average truth judgments around 50% with a peak slightly on the left side. This aligns with the conclusion of the meta-analysis by Dechêne et al. (2010) that the truth effect is the most robust and has the largest effect for ambiguous statements.

Fazio et al. (2019) proposed a theoretical account assuming that repetition increases perceived truth equally for highly plausible, highly implausible, and ambiguous statements. The model assumes a nonlinear mapping of the latent plausibility to the probability of judging a statement as true (see Fig. 1). As a side effect of this transformation, the difference in the probability of “true” responses between repeated and new statements diminishes as plausibility values approach (plus or minus) infinity. Consequently, Fazio et al. ’s (2019) proposal is in line with the fact that the truth effect is absent on the *probability* scale for both highly plausible and implausible statements as plotted in Figs. 5 and 6. Overall, these results indicate that the interaction can be present or absent depending on which scale is considered (Loftus, 1978). Importantly, the exact shape of the assumed link function cannot be tested empirically (Kellen et al., 2020).

In both reanalyses, we scaled the priors for *m*, the parameter governing the magnitude of the truth effect, to be specific for each model variant. This scaling ensured that the implied average truth effect was approximately comparable across models, mitigating the risk that a prior that suits one model may lead to poorer performance for other models. To assess the impact of unscaled priors for *m*, we conducted a sensitivity analysis, which produced mixed and inconsistent results across different datasets, such that the strong evidence for Model 1 against Model 4 disappeared in the reanalysis of the data of Fazio et al. (2019), but remained in the reanalysis of the data of Nadarevic et al. (2018). This finding emphasized that scaling the parameter *m* was crucial for achieving consistent results across different datasets. Additionally, our main analysis primarily relied on relatively restricted priors, namely more informative priors concerning the size of the truth effect. To address whether the priors’ degree of informativeness is effective on the inferences, we conducted another sensitivity analysis to examine the influence of prior restrictiveness by varying the standard deviation of the priors for *m*. The results revealed a consistent pattern across datasets, demonstrating low sensitivity irrespective of whether the priors were restrictive or relaxed.

The proposed Bayesian models can be further validated using other datasets. The main requirements are that the data include statements with a sufficiently large variance in perceived accuracy, that some of the statements are repeated, and that truth judgments are measured with a binary response format. The model can also be generalized to studies investigating other factors that are assumed to influence truth judgments, such as visual contrast (Reber & Schwarz, 1999).

Instead of analyzing aggregated response frequencies, our model accounts for individual biases and dependencies in the data via the subject-level random-effects structure. However, this does not mean that our model can also show how the truth effect varies for the different characteristics of individuals, such as differences in knowledge or memory capacity. Future model versions could integrate subject-level predictors to test whether knowledge protects against the truth effect and whether higher working-memory capacity reduces susceptibility to the truth effect (see Fazio et al., 2015; Schnuerch et al., 2021).

## Conclusion

The proposed Bayesian model offers a formalized theoretical framework and an improved statistical approach for studying the link between the plausibility of statements and the size of the repetition-based truth effect. In contrast to the simulation-based approach, the formal model makes the assumption of a probit link between latent plausibility and truth judgments transparent. This specific, nonlinear scale transformation represents a core assumption of the model because it affects the interpretation of the interaction between plausibility and repetition. Whereas the truth effect might be constant for all statements on the probit scale, it can simultaneously be larger for ambiguous than for highly implausible or plausible statements on the probability scale. Our reanalysis of two datasets corroborated the conclusion by Fazio et al. (2019) that repetition influences perceived truth equally across different levels of plausibility on the probit scale. More generally, our study shows that the specification of precise formal models increases the transparency of the underlying assumptions. Moreover, fitting models directly to data provides stronger conclusions than merely testing certain qualitative predictions derived from simulation-based models.

## Data Availability

The data are available at https://osf.io/q48jv/.

## Footnotes

1 The effect is also referred to as the illusory truth effect, which encompasses truth perception induced by factors beyond repetition. Here, we use the term *truth effect* to specifically denote the repetition-based truth effect.

2 The paper also uses the uncommon parameter label $\mu$ to refer to the standard deviation $\sigma$.

3 Chapman and Chapman (1988) do not mention scale transformations such as the probit-link function.

4 Applying the standard probit-link function to these values shows that $\Phi (1.00)-\Phi (0.60) = 0.12$ while $\Phi (2.14)-\Phi (1.74) = 0.02$.

5 Applying the inverse logit-link function $f^{-1}(.)$ to the same values as before shows that $f^{-1}(1.00)-f^{-1}(0.60) = 0.09$ while $f^{-1}(2.14)-f^{-1}(1.74) = 0.04$.

6 $\text {Normal}(0.11, 0.1)_{\mathcal {I}(0,\infty )}$ assigns 62.2%, $\text {Normal}(0.27, 0.1)_{\mathcal {I}(0,\infty )}$ 86.2%, $\text {Normal}(0.19, 0.1)_{\mathcal {I}(0,\infty )}$ 82.2% and $\text {Normal}(0.38, 0.1)_{\mathcal {I}(0,\infty )}$ 57.7% probability to values in the range from 0.1 to 0.4.

## Declarations

### Open Practices Statement

All data and analysis code are publicly available on the Open Science Framework repository, accessible at https://osf.io/q48jv/.
